# An audit of pregnant women with prosthetic heart valves at a tertiary hospital in South Africa: a five-year experience

**DOI:** 10.5830/CVJA-2012-022

**Published:** 2012-05

**Authors:** B Mazibuko, H Ramnarain, J Moodley

**Affiliations:** Department of Obstetrics and Gynaecology, Nelson R Mandela School of Medicine, University of KwaZulu-Natal, Durban, South Africa; Department of Obstetrics and Gynaecology, Nelson R Mandela School of Medicine, University of KwaZulu-Natal, Durban, South Africa; Women’s Health and HIV Research Group, Nelson R Mandela School of Medicine, University of KwaZulu-Natal, Durban, South Africa

**Keywords:** prosthetic heart valves, anticoagulation, maternal and foetal outcomes

## Abstract

**Background:**

Cardiac disease in pregnancy is a common problem in under-resourced countries and a significant cause of maternal morbidity and mortality. A large proportion of patients with cardiac disease have prosthetic mechanical heart valve replacements, warranting prophylactic anticoagulation.

**Aim:**

To evaluate obstetric outcomes in women with prosthetic heart valves in an under-resourced country.

**Methods:**

A retrospective chart review was performed of 61 pregnant patients with prosthetic valve prostheses referred to our tertiary hospital over a five-year period.

**Results:**

Sixty-one (6%) of 1 021 pregnant women with a diagnosis of cardiac disease had prosthetic heart valves. Fifty-nine had mechanical valves and were on prophylactic anticoagulation therapy, three had stopped their medication prior to pregnancy and two had bioprosthetic valves. There were forty-one (67%) live births, two (3%) early neonatal deaths, 12 (20%) miscarriages and six (10%) stillbirths. Maternal complications included mitral valve thrombosis (*n* = 4), atrial fibrillation (*n* = 8), infective endocarditis (*n* = 6), caesarean section wound haematomas (*n* = 7), broad ligament haematoma (*n* = 1) and warfarin embryopathy (*n* = 4). Haemorrhagic complications occurred in five patients and all five required blood transfusions.

**Conclusion:**

Prophylactic anticoagulation with warfarin in patients with mechanical heart valve prostheses was associated with high rates of maternal and neonatal complications, including significant foetal wastage in the first and early second trimesters of pregnancy. Health professionals providing care for pregnant women with prosthetic heart valves must consistently advise on family planning matters, adherence to anticoagulation regimes and consider the use of prophylactic anticoagulant regimens other than warfarin, particularly during the first trimester of pregnancy.

## Abstract

Women with mechanical prosthetic heart valves (MPHV) are at greater risk of developing complications than those with cardiac disease without MPHV.^1^ The main reason is that MPHV require lifelong anticoagulation to reduce the high risk of associated thrombo-embolic complications.^1^,^2^ In addition pregnancy, being a hypercoaguable state, further increases the risk of thrombo-embolic complications and results in a 35% functional deterioration of MPHV.^2^ It is therefore not surprising that complications associated with cardiac valvular disease in pregnancy carry with them significant mortality and morbidity, particularly in under-resourced countries.^3^-^6^

Prophylactic anticoagulation treatment options for patients with MPHV in pregnancy include warfarin, unfractionated heparin (UH) and low-molecular weight heparin (LMWH).^1^,^7^ These agents are associated with increased maternal and foetal complications, treatment failures, high financial costs and potential teratogenic effects.^1^-^4^,^8^ Warfarin usage in the first trimester of pregnancy, for example is associated with high rates of congenital malformations and foetal losses.^1^-^4^ Therefore many authors suggest that warfarin be replaced by heparin, at least in the first trimester.^1^,^4^-^6^

High rates of treatment failure and mortality with the use of UH have also been reported.^6^ Low-molecular weight or fractionated heparin does not cross the placental barrier and is not reported to have teratogenic potential. Its use for prophylactic anticoagulation therapy may be preferred in pregnancy.^2^,^7^ In addition, LMWH has a longer half-life than UH. However, its use in pregnancy is still controversial due to the lack of adequate clinical trials.^1^,^8^

The majority of pregnant patients with MPHV have been managed at a single tertiary/quaternary facility in Durban by a multidisciplinary team since 2003. It is therefore likely that a single approach to prophylactic anticoagulation was used at this facility. The opportunity was therefore taken to conduct a retrospective chart review of management of patients with MPHV in pregnancy.

## Methods

This was a retrospective study of pregnant patients with MPHV, referred to a tertiary facility for management over a five-year period (January 2005 to December 2009). Ethical and hospital permission were obtained from the appropriate authorities.

At every patient visit, relevant data were captured on a software package (Medicom, Medicom Solutions, India). Baseline data recorded included demographic obstetric information, investigations done and all maternal and foetal complications. All data were captured onto a structured data form.

The policy states that pregnant women with MPHV receive heparin in the first trimester, which is switched to warfarin in the second trimester and then replaced by intravenous heparin at 37 weeks prior to delivery. Heparin is stopped six hours before elective caesarean section (C/S) or induction of labour and re-started 12 hours post C/S or six hours post vaginal delivery if no bleeding complication has occurred. Warfarin, usually 10 mg, is given on the first day, simultaneously with intravenous heparin and the doses adjusted until the INR is 2.5–3 on two consecutive occasions; heparin is then stopped. All patients were treated according to this policy.

Descriptive statistics were used and all results are presented as frequencies, means and percentages.

## Results

Over the five-year study period, 1 021 hospital records of patients with cardiac disease were identified. Sixty-one (6%) had prosthethic valves, mean age 24 (15–45) years. Thirteen (21%) presented in the first trimester; 37 (61%) in the second and 11 (18%) in the third trimester. Fifty-three (85%) were aged ≤ 30 years and 34 (56%) were primigravidae. The demographic and relevant clinical details are shown in Table 1.^9^ In addition to prosthetic heart valve replacements, five patients had associated medical conditions, namely systemic lupus erythematous (*n* = 1), tuberculosis (*n* = 1), insulin-dependent diabetes mellitus (*n* = 1), parathryoidism (*n* = 1) and epilepsy (*n* = 1).

**Table 1:** Baseline Characteristics Of All Pregnant Women With Prosthetic Heart Valves

*Characteristics*	*Number (n = 61)*
Maternal age (years)	
Mean (range)	24 (16–45 )
Age groups (years)	
15–20	17
21–25	23
26–30	13
> 30	8
Parity	
P0	34
P1	17
P2–3	4
P0+1	3
P0+4	1
P1+1	2
Gestational age (weeks) on admission	
< 14	18
14–28	32
28–38	11
History of previous pregnancies	
Miscarriage	6
Intrauterine death	3
Stillbirth (MSB)	2
Neonatal death	2
HIV status	
Negative	43
Positive	16
CD_4_ > 200 cells/ml	14
CD_4_ < 200 cells/ml	2
Declined	2
NYHA functional class	
1	49
11	6
111	4
1V	2

NYHA – New York Heart Association classification.^9^

The dosage details of prophylactic anticoagulation therapy are shown in Table 2 (*n* = 56). Two patients had bioprosthetic valves and were not on any anticoagulation therapy, while three with MPHV had stopped anticoagulation of their own accord prior to pregnancy. Forty-seven patients had isolated mitral valve replacements, 11 had mitral and aortic valve replacements and one an aortic valve replacement for a mean duration of 10 (7–21) years.

**Table 2:** Dosage Details Of Anticoagulation Used By Patients On Presentation At The First Antenatal Visit (*n* = 56)

*Dose of anticoagulation drug*	*Trimester 1 (n = 12) n (%)*	*Trimester 2 (n = 35) n (%)*	*Trimester 3 (n = 9) n (%)*
Warfarin			
2.5 mg	1 (8)	0 (0)	0 (0)
5 mg	6 (50)	23 (66)	5 (56)
7.5 mg	3 (25)	7 (20)	3 (33)
10 mg	0 (0)	1 (3)	1 (11)
40 mg	1 (8)	0 (0)	0 (0)
7.5 mg alt 5 mg	1 (8)	2 (6)	0 (0)
10 mg alt 7.5 mg	0 (0)	2 (6)	0 (0)

**Fig. 1.**
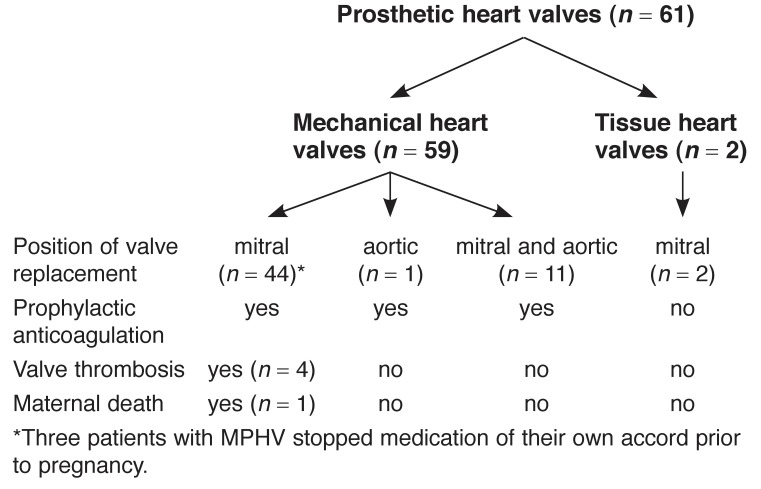
Flow diagram showing position of prosthetic heart valve replacement.

Five patients had thrombotic events. Four with isolated mitral valve prostheses were found on admission to hospital to have thrombosis on echocardiography. The characteristics of four patients with mechanical heart valve thrombosis are shown in Table 3. Three of these patients had repeat MPHV surgery prior to delivery and one at the time of elective C/S. The details of all valve replacement data are shown in Fig. 1.

**Table 3:** Characteristics Of Four Patients With Mechanical Heart Valve Thrombosis

*Patient no*	*Age (years)*	*GA at 1st antenatal visit*	*Parity*	*Age at valve surgery*	*Anticoagulant at 1st antenatal visit*	*Mode of delivery*	*Neonatal outcome*
1	22	21	1	12	Defaulted on therapy prior to pregnancy	NVD	IUD
2	25	36	1	15	Warfarin 5 mg	C/S	Alive
3	21	12	0	11	Warfarin 5 mg	NVD	Alive
4	18	19	0	9	Defaulted on therapy prior to pregnancy	NVD	IUD

IUD = intrauterine death; NVD = normal vaginal delivery; C/S – caesarean section.

The fifth patient with a thrombotic event resulted in a maternal death. The brief details are as follows: a 24-year-old P1G2 presented at 34 weeks gestational age, had a mitral valve replacement and was on warfarin 5 mg daily. Following the stabilisation of her INR at a warfarin dose of 2.5 mg for five to seven days, she complained of severe headache and her INR was 6. The patient was given frozen plasma to stabilise her INR. Shortly thereafter she complained of severe headache and had a low Glasgow coma scale (GCS). CT scan revealed a large left intracerebral bleed. A post mortem C/S was performed and a 2.6-kg live baby with good Apgar scores was delivered.

Echocardiography was performed in all patients. The mean ejection fraction in 57 patients was 55% (range: 34–70) and in four patients < 45%. Five patients showed dilated right ventricle and right atrium. Another patient showed dilated right atrium; one had an ascending aortic aneurysm and another patient had an aneurysm of the aortic root. Two patients had secondary pulmonary hypertension; three had vegetations and were treated for infective endocarditis.

There were 41 live births, two of which ended in early neonatal deaths. There were six stillbirths and 12 miscarriages. The mode of delivery and foetal outcomes are shown in Table 4.

**Table 4:** Delivery Details

*Characteristics*	*Number*	*Percentage*
Anaesthetic		
Spinal	9	22
Epidural	28	68
General anaesthetic	4	10
Live babies (*n* = 41)		
Spontaneous labour		
Delivered vaginally	2 (epidural)	40
Emergency caesarean	3 (1 epidural + 2 GA)	60
Induced labour		
Delivered vaginally	3 (epidural)	50
Emergency caesarean	2 (2 epidural + GA)	50
Elective caesarean (*n* = 31)		
Epidural	21	67
Spinal	9	30
Emergency (GA)	1 (failed spinal)	03
Stillbirths (*n* = 6)		
NVD (spontaneous)	2	33
Induced	4	67
Miscarriages (*n* = 12)		
Warfarin exposure in 1st trimester (NVD)	12	100

GA = general anaesthetic; NVD = normal vaginal delivery.

There were four cases of warfarin embryopathy (three presented for antenatal care in the second trimester and the other in the third trimester). The maternal and foetal characteristics together with the dose of warfarin at admission and sonographic findings are outlined in Table 5. The most common maternal complications during the antenatal period and immediately post delivery are listed in Table 6.

**Table 5:** Congenital Abnormalities Due To Warfarin Embryopathy

*Patient no*	*Maternal age (years)*	*GA (weeks) at 1st antenatal visit*	*Parity*	*Anticoagulant and dose*	*Sonography – congenital abnormalities*	*Foetal outcomes of pregnancy*
1	20	32	1	Warfarin 7.5 mg	Choanal atresia/ microcephaly nasal hypoplasia	ENND
2	22	26	1	Warfarin 5 mg/2. 5 mg	Hydrocephalus, flattening of nasal bone polyhydraminos	SB
3	36	10	2	Warfarin 7.5 mg	Skeletal deformity of spine, nasal hypoplasia, hydrocephalus	SB
4	30	29	0	Warfarin 5 mg	Nasal hypoplasia, mid-facial hypoplasia diaphragmatic hernia	ENND

ENND = early neonatal death; SB = stillbirth; GA = gestational age.

**Table 6:** Maternal Complications During The Antepartum And Postpartum Period

*Complications*	*Number*	*Percentage*
Antepartum		
Epistaxis 2° anticoagulation*	3	5
Atrial fibrillation	8	14
Infective endocarditis	6	10
Valve thrombosis	4	7
Warfarin embryopathy	4	7
Postpartum		
C/S wound haemotomas	7	12
Primary postpartum haemorrhage*	1	2
Postpartum haemorrhage 2° anticoagulation*	3	1

Data expressed as mean (range) or as number (percentage) *Patients requiring blood transfusion.

## Discussion

In this retrospective audit on prosthetic heart valves in pregnancy, the mean age was 24 years and 56% were primigravidae. The low mean age and high number of primigravidae are in keeping with studies originating from other under-resourced countries,^4^,^10^ but not from those of affluent societies.^11^,^12^ A Canadian study reported that the mean age at first antenatal booking was 32 years.^13^ This implies that the severity of rheumatic heart disease may be greater in under-resourced countries, warranting valve replacement at an early age. Furthermore, rheumatic heart disease is uncommon in affluent countries and congenital abnormalities form the majority of cardiac conditions seen in pregnancy.^13^

The high number of pregnancies at an early age in our study may also be due to cultural and socio-economic factors. Such factors may have influenced late booking for maternity care, as 37 (61%) patients attended antenatal care in the second trimester of pregnancy. Late booking for antenatal care and large patient numbers on warfarin throughout the first and second trimesters of pregnancy may indicate that women with cardiac disease do not receive adequate and/or consistent information on family planning, contraceptive services, sexually transmitted infections and the hazards of warfarin therapy in the first trimester.

The challenge for health professionals in under-resourced countries, irrespective of their medical discipline, is to ensure that such information is provided, not only to the individual woman, but also to partners, families and the community at large. Further, it begs the question whether a family planning professional should be attached to cardiac clinics to provide and reinforce appropriate information and prescribe a wide range of contraceptives where necessary. Similar recommendations have been made in the Saving Mothers Reports over the last decade in South Africa.^5^

It is difficult to relate the number of pregnancies to the time from valve replacement. The average age of patients was 24 years, most were primigravidae and the average age of valve replacement was 12 years.

In our audit, the majority of patients were fitted with MPHV. The probable reason for this was that MPHV are cheaper and have greater longevity than bioprosthetic heart valves. However, because of the propensity of MPHV to undergo thrombosis, prophylactic anticoagulation is strongly recommended, if not mandatory.^14^-^16^ Most of our patients with MPHV had either St Jude or Orynx valves. These new-generation MPHV were designed to improve blood flow dynamics, prolong longevity and decrease thrombogenicity. However, our findings and those of others show that these new-generation MPHV are still associated with thrombo-embolism.^17^-^19^

There is therefore no doubt that prophylactic anticoagulant therapy is necessary, but which prophylactic anticoagulation regimen should be used in young women with MPHV, or should women requiring heart valve replacement have bioprosthetic valves inserted prior to pregnancy? Two patients in our study had bioprosthetic valves. Although patients fitted with these valves do not require prophylactic anticoagulation, as they are less thrombogenic than MPHV, the main issues with bioprosthetic valves are their cost, limited lifespan and the possibility of an increased risk of structural valve deterioration (SVD) during pregnancy.^20^ In addition, serious SVD can require re-surgery during pregnancy to replace failing bioprosthetic valves, and all such operations are associated with mortality and morbidity.

Elkayam and Bitar stated that about 50% of women of child-bearing age will require valve replacement owing to SVD seven to 10 years after the original operation.^20^ They also reported SVD in 47% of patients with a history of pregnancy compared with only 14% in non-pregnant patients.^20^ In our study four patients required replacements; three prior to delivery. All three were symptomatic, did not have antenatal care and did not respond to medical treatment. In two of the three cases, the pregnancies ended in intrauterine deaths, while the baby in the third case was born alive. In the fourth patient who had a repeat valve replacement, an elective C/S was planned because she had severe cephalo-pelvic disproportion and a tight mitral stenosis, requiring valve replacement. Both mother and baby did well. These cases illustrate the high perinatal mortality associated with valve replacements during pregnancy.

The morbidity and mortality associated with anticoagulation with warfarin is also high and is probably due to high doses of warfarin in the last two trimesters of pregnancy and the immediate postpartum period. This is illustrated in our study by five cases of thrombosis and the maternal death associated with high doses of warfarin.

There is no current consensus as to the best approach to anticoagulation in pregnant women with MPHV,^19^ as there are no large randomised studies to guide decision making.^18^ In women with MPHVs, the types of anticoagulation that can be used during pregnancy include warfarin, UH and LMWH.

Warfarin, a vitamin K antagonist, crosses the placental barrier, is teratogenic and has been associated with an increased incidence of spontaneous abortion, prematurity, stillbirths and central nervous system developmental disorders.^7^ Furthermore, it would appear that the pivotal period for the risk of foetal congenital abnormalities is between six and 12 weeks of gestation, resulting in a 6–10% risk of embryopathy.^21^,^22^ There are also reports suggesting that warfarin is associated with intracranial foetal bleeding and an increased incidence of stillbirths.^21^,^23^,^24^ Nevertheless because of its ease of use and monitoring, it is commonly prescribed in most under-resourced countries.

Warfarin is also still used in affluent countries. North *et al*. reported high foetal waste rates but low valve thrombotic rates in their series of patients with MPHV using prophylactic warfarin therapy.^25^ If warfarin dosage does not exceed 5 mg daily, the risk of foetal warfarin embryopathy is extremely small.^7^ Vitale *et al*. studied 58 pregnancies in 43 women with MPHV who took warfarin ≤ 5 mg or > 5 mg (target INR 2.5–3.5) until delivery. There were significantly fewer foetal complications in women taking ≤ 5 mg warfarin. It was suggested that warfarin at doses ≤ 5 mg to achieve a therapeutic INR may be safe during the first trimester.^7^

In our study, 29 (50%) patients were on warfarin ≤ 5 mg in the first trimester and did not have congenital foetal anomalies, which was similar to reports from India, Oman and Lebanon.^26^,^27^ However, the four (7%) patients who had embryopathies had warfarin doses of > 5 mg daily.

The risk of miscarriages and stillbirths are also reported to be high in women taking warfarin in the first trimester of pregnancy.^8^ In our study, we had 41 live births, with two resulting in neonatal deaths, 12 miscarriages and six stillbirths. The miscarriages and stillbirths were probably related to high dosages of warfarin and lack of close monitoring of anticoagulant therapy.^21^,^24^ This highlights the need for close and intensive monitoring of warfarin during pregnancy, particularly at the time of switching from one type of anticoagulant to another in the first trimester of pregnancy.

Lack of intensive monitoring at the time of switching from one form of anticoagulant to another is also demonstrated by the high wound haematoma rate in our study, namely seven cases of wound haematomas, three of which required surgical intervention, and one case of broad ligament haematoma, which settled on conservative treatment. There were also four cases of postpartum haemorrhage (Table 6) following the switching of anticoagulants. These cases occurred at C/S, highlighting the need for intense monitoring of coagulation indices at this time. McLintock *et al*. also reported high rates of anteand postpartum haemorrhagic complications using LMWH throughout pregnancy.^11^

A number of studies report that UH and LMWH therapy is safe for the foetus.^21^,^23^ Unfractionated heparin does not cross the placenta and does not have the potential to cause foetal bleeding or teratogenecity. Heparin is generally considered safer than warfarin during pregnancy in terms of embryopathy, however the efficacy of heparin in the prevention of thrombo-embolic complications during pregnancy is contentious. Several reports indicate that its use is associated with high incidence of thromboembolic complications, including fatal valve thrombosis in high-risk pregnant women managed with subcutaneous UH and LMWH therapy.^18^,^19^,^22^,^23^

Chan *et al.* reviewed pregnancy outcomes in women with MPHV and reported thrombo-embolic complications in 3.9% of pregnancies using warfarin only; 9.2% in women who received UH in the first trimester followed by warfarin, and 33% in pregnancies treated with UH heparin throughout pregnancy.^14^ Oran *et al.* reviewed pregnant women with MPHV managed with LMWH and reported complications related to valve thrombosis in 10/81 pregnancies.^28^ Similarly, another review reported thrombotic events in 22% of pregnant women (*n* = 76) managed with LMWH.^29^

More recently, data are emerging that dose-adjusted LMWH (enoxaparin) may be administered safely in pregnancy when there is a dosage adjustment throughout pregnancy to maintain an anti-Xa of 1.0–1.2 U/ml.^30^ There were no thrombo-embolic events in this study of 15 women with MHVP. These reports follow a randomised study in South Africa, comparing UH with enoxaparin, which was stopped prematurely because of two deaths from thrombo-embolism in the enoxaparin group. Anti-Xa levels were measured but no dose adjustment was done.^31^ There is evidence that increasing doses of heparin are required with increasing gestational age because of the increased blood volume and greater renal clearance as pregnancy progresses.^30^

There was one maternal death in our study. Earlier studies originating from under-resourced countries have reported two maternal deaths in 312 patients studied,^4^ one in 229 patients,^32^ and 10 in 480 patients studied.^33^

There was a high rate of maternal complications in our study. Four (7%) patients on warfarin ≥ 5 mg daily developed valve thrombosis in the mitral position. The mitral position is prone to thrombosis and our audit confirms similar findings from other studies.^31^,^34^

Throbo-embolic events and embryopathy are not the only risks that are associated with pregnancies in women with MHVP. Atrial fibrillation, infective endocarditis and C/S wound haematomas were relatively common complications observed in our study.

## Conclusions

This study confirms that the use of warfarin throughout pregnancy carries a significant risk of embryopathy. This risk may be greater with doses of > 5 mg but no definite conclusions can be drawn. In addition, the use of warfarin in the second trimester of pregnancy is associated with significant foetal losses, probably due to poor monitoring and control of warfarin dosages. The switching of warfarin to heparin at the time of delivery may be associated with maternal complications.

Recommendations for management of anticoagulation in pregnant women with MHVP are found in guidelines produced by the American College of Cardiology/American Heart Association.^35^ These guidelines are based on the opinions of experts. An anticoagulation regimen reported by Pieper *et al*. has been modified and shown in Table 7;^8^ and takes into account, the key points of the American guidelines. Large randomised trials of dose-adjusted LMWH are necessary before firm recommendations on an acceptable prophylaxis anticoagulation regimen for prevention of thrombosis of MHVP can be made.

**Table 7:** Anticoagulation Regimen In Pregnant Women With Mechanical Prosthetic Valves

Pre-pregnancy
• Discuss anticoagulation regimen with patient
• Continue warfarin until pregnancy is achieved
• When menstruation is delayed, perform pregnancy test every few days until positive or until menstruation (in order to detect pregnancy at an early stage)
• Give patient and health professional responsible for anticoagulation written instructions about anticoagulation regimen during pregnancy
Sixth to 12th week of pregnancy
• If warfarin daily dose is < 5 mg, consider continuation of warfarin throughout pregnancy
• Alternatively, substitute warfarin with subcutaneous LMWH twice daily
• Adjust LMWH dose to achieve peak anti-Xa levels of 0.7–1.2 U/ml four hours post dose
• If trough levels are sub-therapeutic with therapeutic peak levels, dose three times daily
• Check anti-Xa levels twice a month
13th to 35th week of pregnancy
• Resume warfarin
• Or use LMWH adjusted dose
36th week of pregnancy
• Substitute warfarin with subcutaneous LMWH twice daily
• Adjust LMWH dose to achieve peak anti-Xa levels of 0.7–1.2 U/ml four hours post dose
• If trough levels are sub-therapeutic with therapeutic peak levels, dose three times daily
• Check anti-Xa levels weekly
